# The food web in a subterranean ecosystem is driven by intraguild predation

**DOI:** 10.1038/s41598-021-84521-1

**Published:** 2021-03-02

**Authors:** Andrea Parimuchová, Lenka Petráková Dušátková, Ľubomír Kováč, Táňa Macháčková, Ondřej Slabý, Stano Pekár

**Affiliations:** 1 Department of Zoology, Institute of Biology and Ecology, Faculty of Science, P. J. Šafárik University, Šrobárova 2, 041 54 Košice, Slovakia; 2 Department of Botany and Zoology, Faculty of Science, Masaryk University, Kotlářská 2, 611 37 Brno, Czech Republic; 3 Central European Institute of Technology, Masaryk University, Kamenice 5, 625 00 Brno, Czech Republic

**Keywords:** Ecology, Molecular biology, Zoology, Ecology, Environmental sciences

## Abstract

Trophic interactions of cave arthropods have been understudied. We used molecular methods (NGS) to decipher the food web in the subterranean ecosystem of the Ardovská Cave (Western Carpathians, Slovakia). We collected five arthropod predators of the species *Parasitus loricatus* (gamasid mites), *Eukoenenia spelaea* (palpigrades), *Quedius mesomelinus* (beetles), and *Porrhomma profundum* and *Centromerus cavernarum* (both spiders) and prey belonging to several orders. Various arthropod orders were exploited as prey, and trophic interactions differed among the predators. Linear models were used to compare absolute and relative prey body sizes among the predators. *Quedius* exploited relatively small prey, while *Eukoenenia* and *Parasitus* fed on relatively large prey. Exploitation of eggs or cadavers is discussed. In contrast to previous studies, *Eukoenenia* was found to be carnivorous. A high proportion of intraguild predation was found in all predators. Intraspecific consumption (most likely cannibalism) was detected only in mites and beetles. Using Pianka’s index, the highest trophic niche overlaps were found between *Porrhomma* and *Parasitus* and between *Centromerus* and *Eukoenenia*, while the lowest niche overlap was found between *Parasitus* and *Quedius*. Contrary to what we expected, the high availability of Diptera and Isopoda as a potential prey in the studied system was not corroborated. Our work demonstrates that intraguild diet plays an important role in predators occupying subterranean ecosystems.

## Introduction

A food web represents a network of food chains by which energy and nutrients are passed from one living organism to another. In ecosystems exposed to sunlight, sources of energy originate in autotrophs. In aphotic parts of subterranean environments, colonies of phototrophic organisms were documented only in show caves with artificial light^1^. In cave ecosystems, detritus-based food webs are prevalent, while chemosynthesis is an alternative energy source^2–5^. In the absence of chemoautotrophy, subterranean food webs largely depend on the transport of allochthonous material from the surface^6^. Organic material (remnants of dead plants and detritus) is transported actively or passively into caves by gravitation, ponor streams, or percolating water, while bat guano and animal cadavers or faeces are mostly autochthonous^2,7,8^.

Food webs in caves are simpler and less functionally complex than those in epigean ecosystems due to lower species richness. Subterranean food webs are detritus-based and characterized by bottom-up control^9–11^. Such food webs have been inferred from community composition associated with bat guano^12,13^ and plant roots (see^14^). Studies on trophic interactions between cave-dwelling species are very few^15,16^.

The primary consumers of organic material deposited in caves (guano deposits, rotten wood, etc.) are microorganisms, such as Bacteria, Archaea and Fungi^2,8^. Microbivores (some Acari and most Collembola) are attracted by colonies of such decomposers. Invertebrate detritivores (e.g. Oligochaeta, Isopoda, Diplopoda, some Coleoptera) consume decomposing organic material, enhance the transfer of nutrients through fragmentation and foster microbial activity, which in turn increases the rate of organic matter decomposition^9^. Detritivorous and microbivorous invertebrates are preyed upon by several predators. In caves, predators are mostly represented by Chilopoda, Araneae, Pseudoscorpiones, carnivorous Acarina, and some Coleoptera^2,13,14^. Trophic linkages in subterranean food webs indicate a trend toward generalist strategies, similarly as in soil food webs^17,18^. Thus, subterranean food webs are often truncated with few or no strict predators at the top^9^.

Investigation of trophic interactions among arthropods can be performed using several approaches^19^. In caves, a number of studies have revealed dynamics at higher taxonomic levels using the signatures of stable isotopes^20–22^. Modern molecular methods have not been used. Yet these methods offer an exclusive opportunity to identify food items with high taxonomic precision, even from the gut of very tiny predators^23^. Thus, this method can unveil cryptic feeding and provide data for subsequent reconstruction of food webs^24–27^.

**Figure 1:**
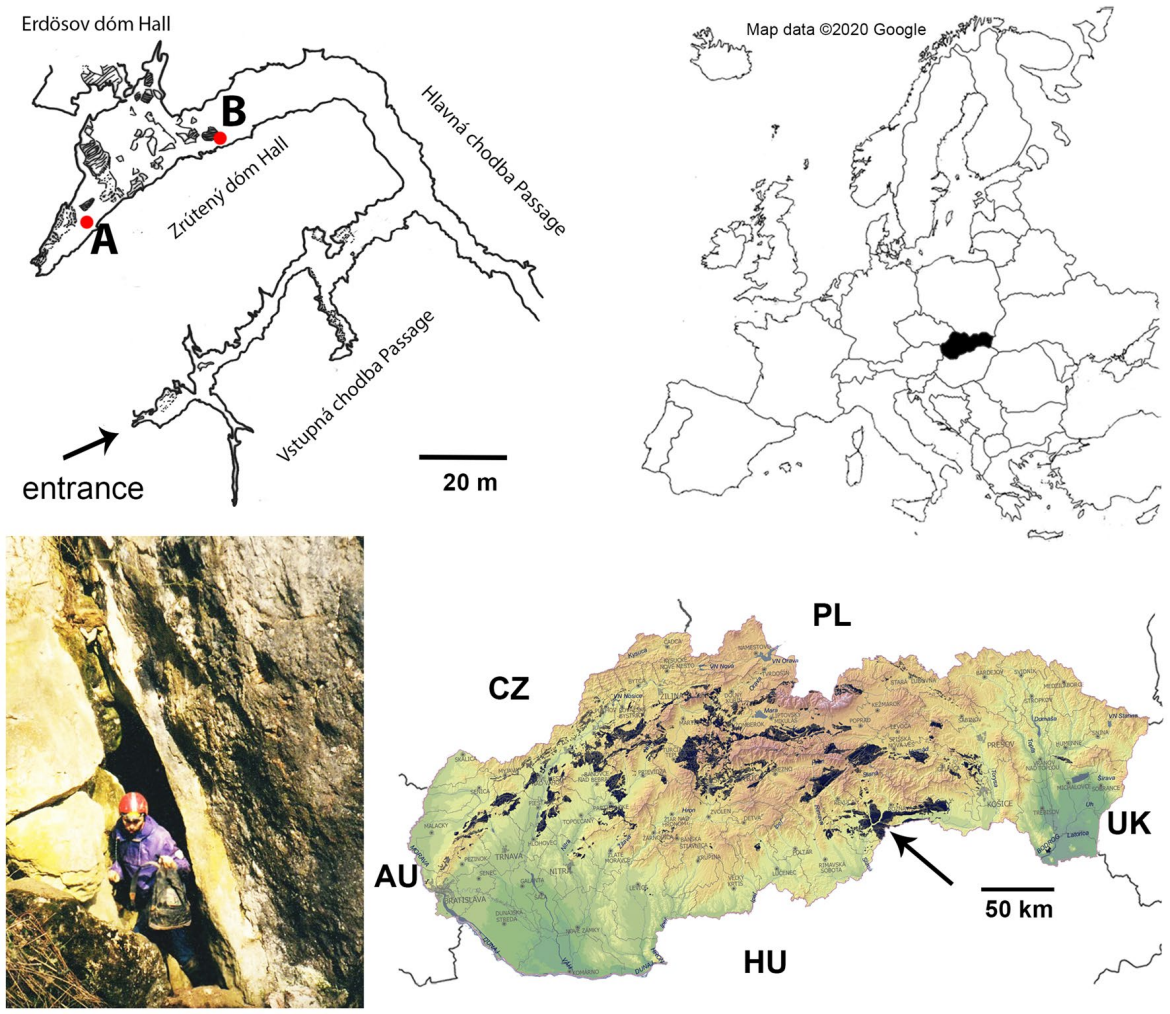
Location of Ardovská Cave in Slovakia and sampling cave sites. The section of upper cave level redrawn by A. Parimuchová from the original map^72^. Red points: (**A**) chamber behind Zrútený dóm Hall; (**B**) Zrútený dóm Hall. The map of Europe adopted from www.google.com, the map of Slovakia displaying karst areas (dark spots) created by the Slovak Museum of Nature Protection and Speleology, digitally processed by P. Gažík. The arrow indicates location of Ardovská Cave. The cave entrance photo taken by Ľ. Kováč. All figures processed using Adobe Photoshop CS6 (www.adobe.com).

In the present study, we aimed at investigating a food web using molecular analyses of predator gut-content in the subterranean ecosystem of the Ardovská Cave in Slovakia (Fig. 1). Thanks to the intensive research previously conducted on the invertebrate community in this cave^28–30^, the taxonomic composition and trophic classification of the cave-dwelling species is well known. The relatively dense population of the palpigrade *Eukoenenia spelaea* enabled us to include this rare species into the study as well. We expected a high level of intraguild interactions among predators, induced by low habitat heterogeneity combined with a lack of primary producers and low species diversity of invertebrates. We considered cave predators to feed unselectively on a wide variety of prey items. We hypothesised that *Eukoenenia* is a true predator despite a previous observation of Cyanobacteria in its alimentary tract^31^. In this study we aimed at (1) an analysis of the predator food web of Ardosvká Cave, and (2) including the feeding habit of *Eukoenenia spelaea*, a very rare cave arachnid occupying a model cave in a stable population.

## Results

### Sequencing output

In the gut of 105 individuals belonging to five predator species (Table 1), 20 prey species were identified (Table S1). We obtained 11,697,054 paired-end reads as the sequencing output (10,957,042 remained after the paired-end reads were merged). Nearly 50% of the reads (5,265,956 seq.; 48%) were informative (predator and prey sequences). The sequences were clustered into 87 different operational taxonomic units (OTU), 13 of which (3,644 sequences; 0.03%) showed obvious contamination and were removed. Fungi (10 OTUs; 246 seq.), algae (1 OTU; 3 seq.), protozoans (4 OTUs; 69 seq.), and nematodes (3 OTUs; 3,856 seq.) were found rarely in the guts and were excluded from the subsequent analyses, as they did not represent prey. Prey was represented by 56 OTUs assigned to 11 orders and 22 families (Table S1).

**Table 1:** List of invertebrates collected in the Ardovská Cave, Slovakia (for collecting sites, see Fig. 1).

Order/family	Species	Stage	No. of inds	Collecting site		Status	Body length (mm)
				Cave part	Microhabitat		
**Palpigradi**	*Eukoenenia spelaea* (Peyerimhoff, 1902)	Indet	18	B*	Sediment	P	0.7–1.32
**Acari**							
Parasitidae	*Parasitus loricatus* (Wankel, 1861)	Ad	10	B*	Rotten wood	P	1.2–1.45
		Juv	29	B*	Rotten wood	P	0.4–1.25
Veigaiidae	*Veigaia* sp.	Ad	2	B*	Rotten wood	P	0.7
		Juv	1	B*	Rotten wood	P	0.6
Macrochelidae	Indet		3	B*	Rotten wood	P	0.7–0.85
**Araneae**							
Linyphiidae	*Porrhomma profundum* (Dahl, 1938)	Ad	4	B*	Rotten wood	P	1.95–2.5
Linyphiidae	*Centromerus cavernarum* (Koch, 1872)	Ad	5	B*	Rotten wood	P	1.4–1.72
Linyphiidae		Juv	21	B*	Rotten wood	P	0.7–2.25
**Coleoptera**							
Staphylinidae	*Quedius mesomelinus* (Marsham, 1802)	Ad	18	A	Stalagmites and sediment	P	9–13.5
**Acari**							
Oribatida							
Damaeidae	*Kunstidamaeus lengersdorfi* (Willmann, 1932)		7	Entrance passage	Rotten wood	c	0.55
**Isopoda**							
Mesoniscidae	*Mesoniscus graniger* (Frivaldszky, 1865)		19	B*	Sediment	c	≤ 20
**Diplopoda**						c	
Trichoplydesmidae	Indet		4	B*	Sediment	c	3
Trachysphaeridae	*Trachysphaera costata* (Waga, 1857)		13	B*	Sediment	c	2.5–5.5
**Collembola**							
Arrhopalitidae	*Pygmarrhopalites aggtelekiensis* (Stach, 1930)		5	A	Stalagmites	c	1.4
Neelidae	*Megalothorax minimus* Willem, 1900		5	A	Water pools	c	0.5
Entomobryidae	*Heteromurus nitidus* (Templeton, 1835)		20	B*	Sediment	c	2
Entomobryidae	*Pseudosinella aggtelekiensis* (Stach, 1929)	Indet	10	A	Stalagmites	c	2.1–2.4
Onychiuridae	*Deuteraphorura kratochvili* (Nosek, 1963)	Indet	4	*	Sediment	c	1.5–2.2
Tullbergiidae	*Mesaphorura jirii* Rusek, 1982	Indet	15	*	Sediment	c	0.42
Isotomidae	*Parisotoma notabilis* (Schäffer, 1896)	Indet	6	*	Sediment	c	1
Isotomidae	*Folsomia candida* Willem, 1902	Indet	3	*	Sediment	c	1.5–3
**Diptera**							
Nematocera						c	
Sciaridae	*Bradysia forficulata* (Bezzi, 1914)	Ad	3	Entrance	Rotten wood	c	≤ 8
Trichoceridae	*Trichocera regelationis* (Linnaeus, 1758)	Ad	2	Entrance	Walls	c	≤ 13
Brachycera						c	
Phoridae	*Triphleba antricola* (Schmitz, 1918)	Ad	3	Entrance	Rotten wood	c	≤ 5

P = predator; c = prey; ad = adult; juv = juvenile;* = occurrence throughout the cave (for more details, see Fig. 1). The body lengths of prey species are based on literature data. In Diptera, the maximal length of larvae is shown.

### Trophic niche

**Figure 2:**
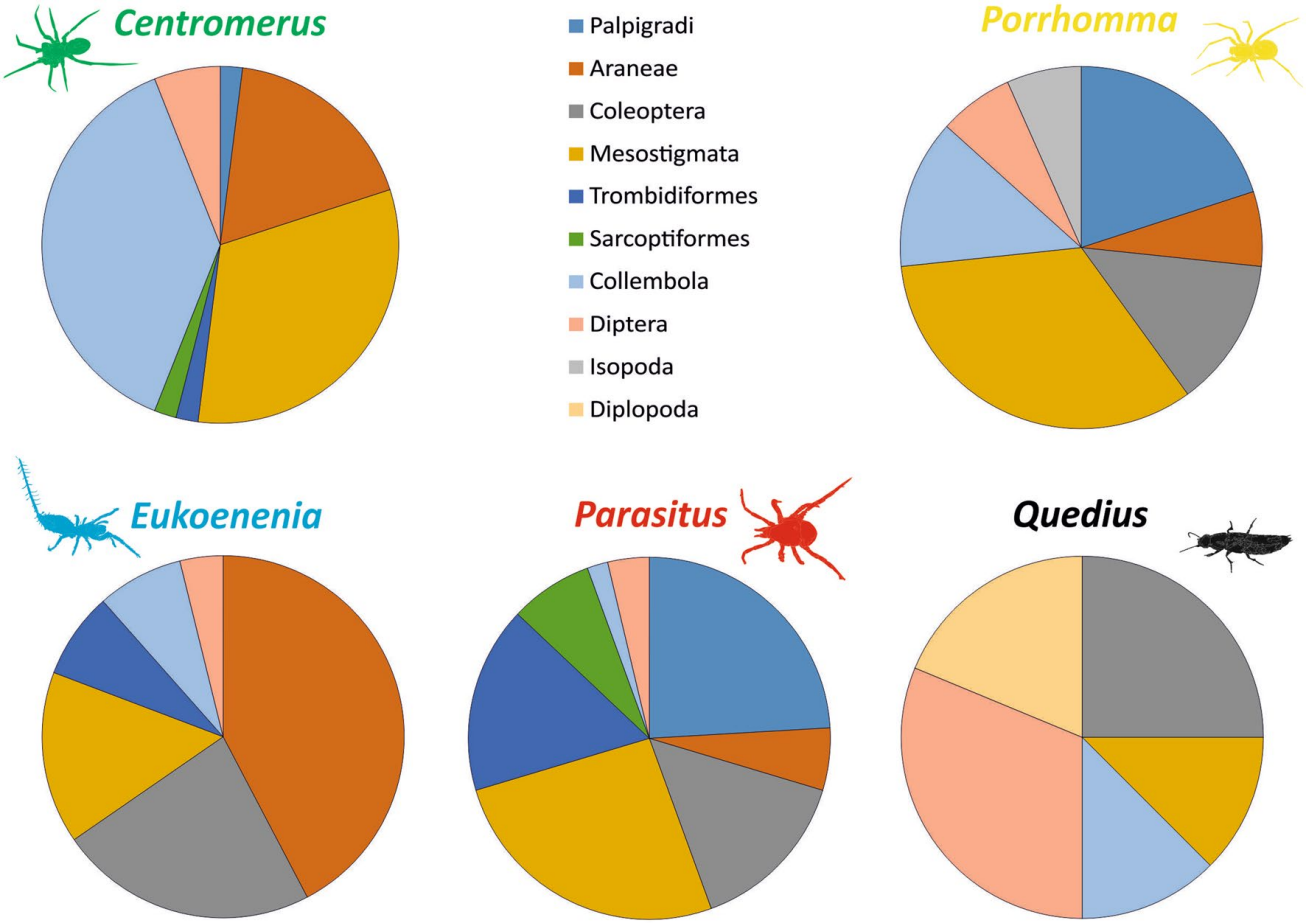
Pie charts showing the proportions of 10 prey types found in the guts of the five predators considered in the gut content analysis. Drawings were created by S. Pekár.

The five predatory species from aphotic cave parts were: a mite (*Parasitus loricatus* (Wankel, 1861)), a beetle (*Quedius mesomelinus* (Marsham, 1802)), a palpigrade (*Eukoenenia spelaea* (Peyerimhoff, 1902)), and two spiders (*Porrhomma profundum* (Dahl, 1938), (*Centromerus cavernarum* (Koch, 1872)). The gut of the *Porrhomma* spiders mainly contained the DNA of mites, palpigrades, beetles, and springtails (Fig. 2). *Centromerus* spiders mainly had the DNA of springtails, mites, and spiders. The gut of *Eukoenenia* palpigrades contained mainly spiders, followed by beetles. The *Parasitus* mites mainly had the DNA of mites, palpigrades and beetles, and the *Quedius* beetles mainly had the DNA of flies, followed by millipedes, mites, and springtails, in their guts (Table S1).

**Figure 3:**
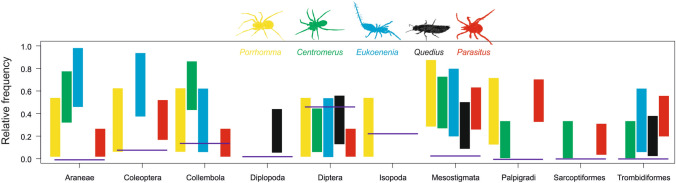
Comparison of the relative prey availabilities of 10 orders (horizontal line) and 95% confidence intervals (bars) of their probability of being found in the guts of the five considered predators. Confidence intervals were estimated using GLM-b. Intervals for zero probabilities were omitted. Drawings were created by S. Pekár.

A different picture emerged when comparing the proportions of prey in the guts of predators with their availability in the environment. In all five predators certain prey sequences were represented more than others, regardless of prey availability (Fig. 3). Most of the predators had sequences of spiders, palpigrades, coleopterans, mites (Mesostigmata, Trombidiformes, Sarcoptiformes), and collembolans significantly more frequently in their gut than sequences of the other available prey. In contrast, the sequences of dipterans were represented in all predators less frequently.

The trophic niches of these five predators were moderately narrow to wide. *Centromerus* had the narrowest niche (*B*_*A*_ = 0.28), followed by *Eukoenenia* (0.30), *Quedius* (0.38), *Porrhomma* (0.44), and *Parasitus* (0.49). The trophic niches of *Porrhomma—Parasitus* and *Centromerus—Eukoenenia* overlapped the most (*O* > 0.57), while the overlap was lowest between *Parasitus—Quedius.* For all other predators the overlap was moderate (Table 2).

**Table 2:** Overlap of the trophic niches of five predators estimated by Pianka’s index.

	*Centromerus*	*Eukoenenia*	*Parasitus*	*Porrhomma*
*Eukoenenia*	0.573	–	–	–
*Parasitus*	0.369	0.309	–	–
*Porrhomma*	0.397	0.353	0.846	–
*Quedius*	0.419	0.399	0.129	0.332

**Figure 4:**
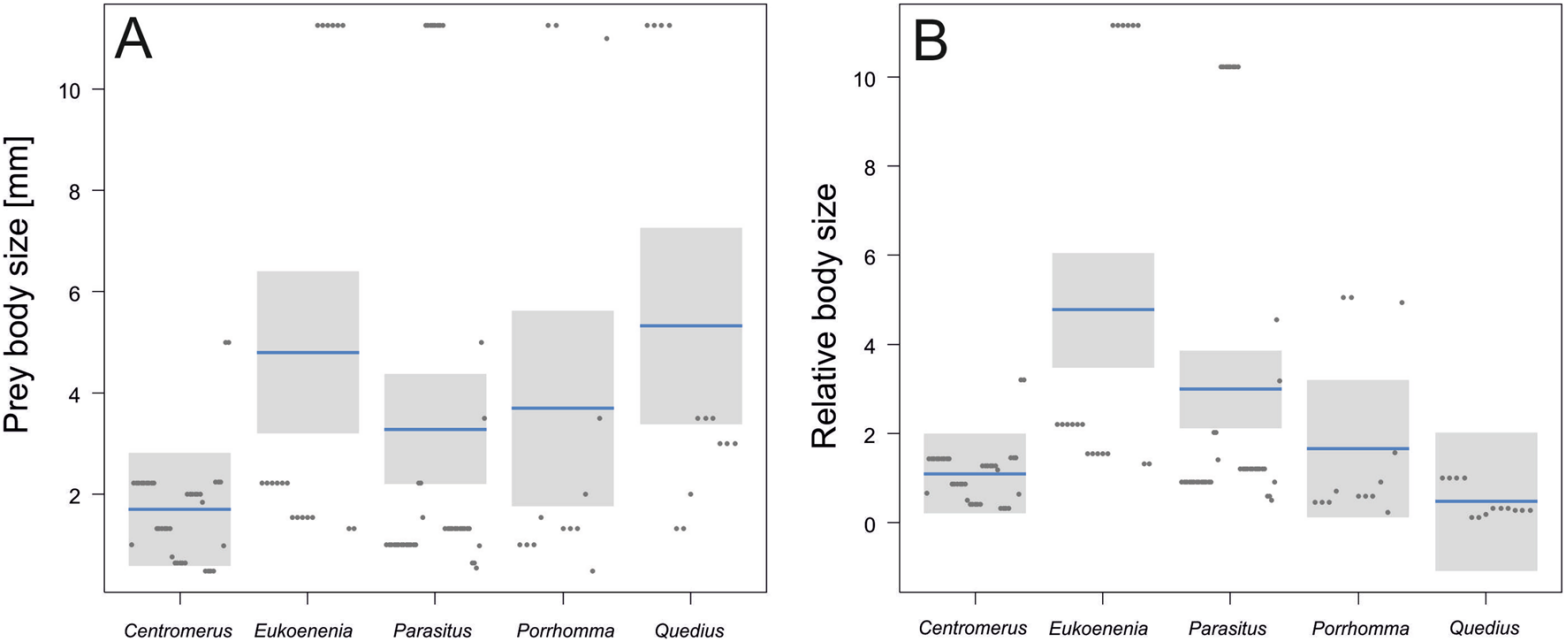
Comparison of the absolute prey size (**A**) and relative prey size (**B**) among the five predators considered in this study. Relative prey size was estimated as average prey size/average predator size. Horizontal lines are estimated means and boxes are 95% confidence intervals. Measurements are jittered.

There was a significant difference among predators in terms of absolute (LM, F_4,120_ = 3.9, P = 0.0049) and relative (LM, F_4,120_ = 7.5, P < 0.0001) prey body size. *Eukoenenia* had sequences of the largest prey. Absolute prey body size decreased from *Quedius* (and *Eukoenenia*) followed by *Porrhomma*, *Parasitus*, and *Centromerus* (Fig. 4A). The former two species had the highest prey-size-niche overlap. Relative prey size then decreased from *Eukoenenia* through *Parasitus*, *Porrhomma*, *Centromerus*, and *Quedius* (Fig. 4B).

### Intraguild predation (IGP)

**Figure 5:**
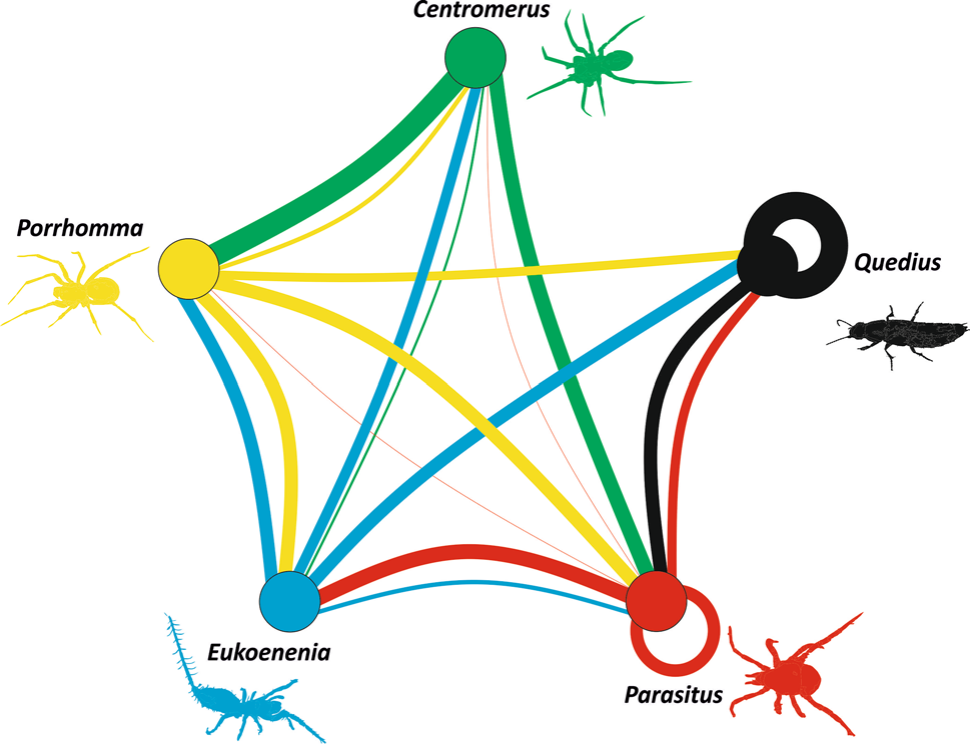
Trophic food web among the five predators considered in the analysis. Width of lines corresponds to the proportion of predator–prey interactions. Drawings were created by S. Pekár.

Predatory arthropods represented 12.2% (N = 757) of available prey, yet, IGP was significantly more frequent than expected in all five predators. According to the Binomial test, IGP was significant in *Eukoenenia* (87.5%, P < 0.0001), followed by *Centromerus* (81.3%, P < 0.0001), *Parasitus* (80.0%, P < 0.0001), *Porrhomma* (62.5%, P = 0.001), and *Quedius* (37.5%, P = 0.0080). Consequently, the food web showed high connectivity, with *C* = 0.80 (Fig. 5). Intraspecific consumption was confirmed only in *Parasitus* (44.0%) and *Quedius* (25.0%).

## Discussion

Here we aimed at an analysis of the food web within the guild of cave predators, based on comprehensive molecular gut content analysis. The diet composition was used to define the trophic niches of five frequent predators occupying aphotic cave parts. The width and overlap of the trophic niches helped to reveal trophic interactions among these predators.

There is a lack of data on the diets of these predators, particularly the two spider genera *Centromerus* and *Porrhomma*^32^. Generally, epigean linyphiid spiders are euryphagous, and collembolans, homopterans, and dipterans are an important part of their diet^33^. *Centromerus* and *Porrhomma* congeners are euryphagous as well, but they probably compete for different prey resources in the studied cave. We found that *Centromerus* fed mostly on collembolans, while *Porrhomma* fed predominantly on mites.

Very little is known about the feeding habits of palpigrades, and recent studies have provided controversial evidence^31,34^. Carnivory is expected in Palpigradi based on the morphology of the mouth-parts (the relatively large chelicerae), which form pincer-like structures with crossed elongated tips^35,36^. Our study revealed a relatively broad spectrum of prey taxa that supports carnivory in *Eukoenenia spelaea*. Moreover, recent observation of *Eukoenenia* hunting small prey (a neelid collembolan) on the surface of a water pool^34^ provides evidence of a predatory habit. Surprisingly, spiders, beetles, and mites represented a considerable part of *Eukoenenia*’s diet. The predation of *Eukoenenia* on adult invertebrates (larger than palpigrades) is questionable, but feeding on eggs or juvenile stages of these arthropods is probable, as suggested by Wheeler^37^. Scavenging is another possible, but less likely explanation for the presence of spider, mite, and beetle DNA in the gut of *Eukoenenia*. The presence of Cyanobacteria in their gut in a former study^31^ may indicate an alternative food source or a case of secondary predation^38^. Accidental consumption of Cyanobacteria attached to food (body of prey) could also be the case. We did not detect any Cyanobacteria or Algae in palpigrades; however, Fungi were found in two individuals.

The limited data on the trophic ecology of *Parasitus* congeners come from studies on the potential biocontrol species *Parasitus bituberosus* (Karg, 1972). It is euryphagous, preying mainly on nematodes, mites, and flies^39,40^. Similarly, our results show that *Parasitus loricatus* is euryphagous, too, feeding predominantly on other mites. We also detected nematodes of the order Rhabditida (Rhabditidae) in these mites. Free-living bacteriophagous nematodes are abundant in the sediment of the Ardovská Cave (Renčo, pers. comm.) and could serve as a food for these mites. On the other hand, several representatives of this nematode order are saprophagous, free-living facultative parasites of insects^41^, with some species infecting arachnids^42^. Due to the ambiguity of the detected nematodes (prey versus parasite) in our study, we did not consider them as prey.

*Quedius* beetles were also found to be euryphagous. Despite feeding on a large variety of prey, they fed mainly on flies, resulting in a very low niche overlap with the other predators. Their prey selection was likely influenced by body size and the agility of the prey. Dipteran larvae represent easily accessible prey, especially for beetles and mites in the studied cave. Less active species or life stages of invertebrates can be preyed upon by *Quedius* beetles, which are characterised by high searching activity^43^. A similar feeding preference for less motile prey (such as millipedes) has been reported for staphylinid beetles^44^.

Prey choice differed in the studied predators, as was indicated by the overlap of trophic niches. A comparison of prey exploitation and their availability supports the knowledge that predators often select prey according to its nutritional composition in order to optimize their demands^45^. This has been suggested to occur in habitats with nutrient deficiency and low diversity with respect to potential prey^46^. Diptera and Isopoda directly associated with bat guano represented the most available potential prey in the studied cave. Despite the effort when collecting to gain an objective picture of the composition of cave arthropod assemblages, we are aware of possible sampling bias in estimating the food availability for predators.

Although the DNA in the alimentary tract of the predators was thoroughly identified, the interpretation of the results is a bit problematic. Secondary predation as well as scavenging could be a significant source of errors in the molecular detection of prey in predators^25^. Taking into account the number of particular prey sequences detected in predators, we do not presume their origin from secondary predation. Primary prey can be detected in a secondary predator only very soon after consumption of the primary predator. Thus, the probability that a considerable portion of the detected prey came from secondary predation is low. As we cannot distinguish predation from scavenging, we interpreted the origin of allochthonous DNA in a predator’s body based on knowledge of their mouth-parts morphology and in situ behavioural observations. Thus, we explained the presence of prey DNA in predators by predation. A high frequency of IGP is possibly a result of the higher frequency of encounters among active predators in habitats of low heterogeneity^47–49^, such as caves.

In the present study, conspecific DNA was detected in mites and beetles. Intraspecific predation (cannibalism) and scavenging are possible explanations for such results. We assume it was rather cannibalism in both *Parasitus* and *Quedius*, as we observed predatory behaviour while dead prey was rejected. There are many records of cannibalism among arachnids^50–53^. Both IGP and cannibalism supplement a nitrogen‐poor diet^54–56^. A mixed feeding strategy composed of cannibalism and interspecific predation provides high quality food^57^ in which conspecifics may serve as bio-accumulators, concentrating valuable nutrients in their bodies.

Based on the present results, we conclude that the five arthropod predators occurring in the studied cave are all euryphagous, feeding on a wide variety of prey. The different trophic niche overlap of particular predators suggests different prey preferences. The *Quedius* beetle mainly exploits Diplopoda and Diptera larvae associated with bat guano, while spiders, as “sit-and-wait” predators, feed on more motile prey, such as Acari and Collembola. The prey spectrum of *Parasitus* mites and *Eukoenenia* palpigrades is comparable with that of spiders. The studied carnivores considerably exploit other predators as interspecific prey, and in the case of cannibalistic mites and beetles also as intraspecific prey. The high proportion of intraguild predation may represent an important trait of ecosystems lacking phototrophs. The feeding ecology of *Eukoenenia* remains poorly known. In situ observations and feeding experiments may help to reveal the proportion of hunting and scavenging in this mysterious subterranean animal.

## Materials and methods

### Study site

The Ardovská Cave is a publicly inaccessible natural limestone cave situated in the Slovak Karst, in the Western Carpathians Mountains (314 m a.s.l., N 48° 52.2367′ E 20° 42.0816′) (Fig. 1). The only cave entrance is a narrow, two-metre high fissure behind which the cave continues down by small passages to larger spaces of the upper cave level, where the study was carried out. The entrance section thus prevents air circulation and determines the very stable microclimate inside the cave. The mean temperature of the upper horizontal level is ~ 10.7 °C and air humidity ~ 100%^30^. The presence of several guano heaps and wood remnants along this level suggest meso-eutrophic conditions^28,30,58^. The hygropetric microhabitat (speleothems with percolating water) is typical of the back parts of the upper cave level, but predators are rarely observed there. A water stream appears in the lower cave level after heavy rainstorms. The cave is inhabited by several troglobionts, most of them Western Carpathian endemics^28–30^.

### Sampling

For molecular investigation, five predatory species and 16 frequent potential prey species were hand-collected throughout the upper cave level (Table 1). Among the parietal fauna, only dipterans, with larvae living in rotten organic material, were included. All specimens (predators and prey) were collected using a brush and tweezers during one visit in autumn 2017 and five visits in 2018 (all year round) in the upper level of the cave, in the section from the entrance passage to its back parts. The collected specimens were immediately placed in 96% ethanol and stored at – 18 °C until analyses. As the population of *E. spelaea* inhabiting the Ardovská Cave is unique and its reproduction rate is unknown, a maximum of eight large individuals per visit were captured in order to minimize the effect on the population size.

In order to estimate the availability of potential prey, we used abundance data obtained during the monitoring of the cave invertebrates in April–October 2010 (Table S2). Five sites were selected and a combination of several collecting methods was used to cover the diversity of terrestrial arthropods in the cave: (1) pitfall trapping—five traps (150 mL plastic jar) were exposed at each site for 5 months, filled with one of three types of fixation liquids (95% denaturated ethyl alcohol—two traps, 4% water solution of formaldehyde – two traps, and a mixture of ethylene glycol and beer in a 1:1 ratio—one trap); (2) baiting—100 cm^3^ of sterilized wood shavings were exposed for 5 months near the traps at each study site; (3) extraction of organic material (wood remains, bat guano, baits) in a high-gradient apparatus^59^; and (4) visual search and hand collection using tweezers, brushes, and pooters was performed during two cave visits. Organic material and baits were transported to the laboratory for extraction immediately after the sampling.

### DNA isolation and prey detection

DNA was isolated from the predators and their potential prey using the E.Z.N.A. Tissue DNA kit (Omega Bio-tek) according to the manufacturer’s protocol. Altogether, 81 individuals of five predator species were used in this study: *Eukoenenia spelaea* (N = 15), *Porrhomma profundum* (N = 9), *Centromerus cavernarum* (N = 16), *Parasitus loricatus* (N = 25), and *Quedius mesomelinus* (N = 16). The DNA concentration was measured using Qubit and diluted to reach the optimal concentration of 10–20 ng/µl.

The cytochrome oxidase I gene was amplified using LCO1490 and HCO2198 primers^60^ to obtain reference sequences for the predators and their potential prey species (Table S3). The PCR reaction mixture consisted of 0.7 µL of forward and reverse primers (10 µLM), 0.2 µL of PCRBIO HiFi polymerase, 4 µL of 5X HiFi buffer, 9.4 µL of ultraclean water, and 5 µL of DNA. PCR was conducted under the following conditions: initial denaturation at 95 °C for 5 min; 35 cycles of 95 °C for 30 s, 46 °C for 30 s as an annealing temperature, 72 °C for 75 s; and a final extension at 72 °C for 5 min. The PCR products were sequenced on an ABI Prism 3130 Genetic Analyzer (Applied Biosystems). The sequences are deposited in GenBank (accession numbers: MN906450–MN906467). Reference sequences (at least at the genus level) for three taxa—*Mesoniscus graniger, Trachysphaera costata, Megalothorax minimus*—were found in NCBI, but we failed to obtain any COI sequence for *Mesaphorura jirii.*

To maximize the detection of prey species in predator guts, PCR was performed with MiteMiniBar primers^61^ and the Multiplex PCR Kit (Qiagen). Nextera overhang adaptors (Illumina) were attached to these primers. The primers were tested against all potential prey types to check whether the primers allowed the amplification of all these prey types. All but two species (*E. spelaea* and *M. jirii*) were amplified using the primers. The PCR reaction mixture consisted of 5 µL of DNA, 0.8 µL of the forward and reverse primers (10 µM), 10.6 µL of Multiplex master mix, 1.8 µL of Q Solution, and 3 µL of ultraclean water. The PCR conditions were as follows: initial denaturation at 95 °C for 15 min; 35 cycles of 94 °C for 30 s, 50 °C for 90 s as an annealing temperature, 72 °C for 90 s; and a final extension at 72 °C for 10 min. The PCR products were detected on 2% GoodView-stained agarose gels. After the primer tests, PCR was performed with DNA isolated from the predators under the same conditions. Library preparation (PCR II with Nextera indexes, DNA concentration measurements on Qubit, the pooling of samples, purification using Agencourt AMPure X beads) and paired-end read sequencing with NextSeq 500/550 Mid Output Kit v2.5 (300 Cycles) were performed on an Illumina NextSeq 500 instrument at the CEITEC Genomic Core Facility (Brno, Czech Republic).

After sequencing, the reads were split according to the combination of their indices. The data were processed using the Galaxy platform (www.usegalaxy.org). Paired-end reads were merged using fastq-join^62^, with a maximum difference of 10%. The adapters and primers were trimmed using cutadapt^63^ with a 0.15 error rate and two-thirds of the primer as the minimum overlap option. The data were then filtered by quality (Q30), filtered by length to remove reads that were too short and too long, and then collapsed. All OTUs were manually checked against OTUs which were 100% similar to the reference sequences in BOLD, while the OTUs containing two parts of different organism were considered as chimeric. Rare haplotypes (containing less than two identical reads) were excluded, and sequences containing insertions or deletions causing reading frame shifts and chimeric sequences were removed. The remaining sequences were clustered into OTUs with 4 bp difference using swarm^64^, and chimeric sequences were completely removed. Each OTU was compared to the NCBI database (https://blast.ncbi.nlm.nih.gov/Blast.cgi) using megablast, to the BOLD database (https://www.boldsystems.org/), and to the reference sequences obtained from invertebrates collected in the Ardovská Cave. The prey was assigned to a taxonomic level based on the percentage similarity to the reference sequences.

As the MiteMiniBar primers did not attach to *E. spelaea* DNA, specific primers which would detect *Eukoenenia* as the prey in other predators were designed (Eukoe28SF274: 5´-ACTGAGCGGGAGCAAGGGTGGTTTGC, Eukoe28SR415: 5´- GTGACCGACCTACTCGCCGCAGATG) based on the 28S rRNA gene, which was amplified using 28SF2 and 28S3DR primers^65^ and sequenced as described above. All predators (except *E. spelaea*) were screened using these primers. The PCR reaction mixture consisted of 5 µL of DNA, 0.7 µL of forward and reverse primers (10 µM), 0.2 µL of PCRBIO HiFi polymerase, 4 µL of 5 × HiFi buffer, and 9.4 µL of ultraclean water. The PCRs were performed under the following conditions: initial denaturation at 95 °C for 5 min; 35 cycles of 95 °C for 30 s, 60 °C for 30 s as an annealing temperature, 72 °C for 75 s; and a final extension at 72 °C for 5 min. The PCR products were detected on 2% GoodView-stained agarose gels, and ambiguous PCR products were sequenced on an ABI Prism 3130 Genetic Analyzer (Applied Biosystems).

### Analyses

The standardized Levins’ index (*B*_*A*_) of niche breadth^66^ was used to calculate the breadth of the realised trophic niche of all predators at the order level of prey. Values of *B*_*A*_ higher than 0.6 indicate a wide niche, while values below 0.4 indicate a narrow niche^67^. Pianka’s index (*O*)^68^ was used to calculate niche overlap among the predators, with values close to one indicating the highest niche overlap.

Linear models (LM) were used to compare absolute and relative prey body sizes among the predators^69^. The body sizes of the predators were measured under a binocular stereomicroscope and ocular ruler prior to DNA isolation. For specification of the body length of the prey species, we used literature data on average body size, estimated from several developmental stages for each species. Relative prey size was estimated as average prey size/average predator size. GLM with binomial errors (GLM-b) were used to estimate 95% confidence intervals for each of 10 prey types in the guts of five predators^69^. A binomial test was used to compare the proportion of predators positive for a certain prey with the relative frequency of prey availability.

All statistical analyses were performed in the R environment^70^. To visualise the results, we used the visreg package^71^.

## Supplementary Information

Supplementary Information.

## Data Availability

DNA sequences are deposited in GenBank (Accession numbers: MN906450–MN906467).
