# Supplementary material for: The food web in a subterranean ecosystem is driven by intraguild predation
Source: Sci Rep. 2021 Mar 2;11:4994. doi: 10.1038/s41598-021-84521-1 (PMC7925651; doi:10.1038/s41598-021-84521-1)
Supplement: Supplementary file 1 — Supplementary Information. [file 41598_2021_84521_MOESM1_ESM.pdf]

The food web in a subterranean ecosystem is driven by intraguild predation

Andrea Parimuchová, Lenka Petráková Dušátková, Ľubomír Kováč, Táňa Macháčková, Ondřej Slabý, Stano Pekár

Table S1. List of DNA sequences of prey detected from the guts of five predators. No. # seq. = number of prey sequences in each MOTU per individual; Predator seq. = number of predator sequences. Presence of DNA of *Eukoenenia* was detected by PCR in all individuals of predators; therefore, a sequence number is not available.

Predator	Order	Family	Genus/Species	No. of seq.	Predator seq.
Porrhomma profundum	Mesostigmata	Laelapidae		1943	256653
		Parasitidae	*Parasitus loricatus*	86	
	Arthropoda unidentif.			25	
	Palpigradi	Eukoeneniidae	*Eukoenenia spelaea*	-	
	Isopoda			19	88333
	Collembola	Entomobryidae	*Heteromurus nitidus*	10	
	Palpigradi	Eukoeneniidae	*Eukoenenia spelaea*	-	
	Mesostigmata	Parasitidae	*Parasitus loricatus*	193	42201
			unidentif.	5	
				0	48737
				0	23157
	Mesostigmata	Parasitidae	*Parasitus loricatus*	343	61800
	Araneae	Linyphiidae	*Centromerus cavernarum*	10	
	Collembola	Neelidae	*Megalothorax*	3	
	Palpigradi	Eukoeneniidae	*Eukoenenia spelaea*	-	
	Mesostigmata	Laelapidae		2	21234
				0	28947
	Diptera	Phoridae	*Triphleba antricola*	498	55186
	Coleoptera	Staphylinidae	*Quedius mesomelinus*	2	
Centromerus cavernarum	Mesostigmata	Laelapidae		9732	40362
		Parasitidae	*Parasitus loricatus*	59	
			unidentif.	4	
		Ameroseiidae?		9	
	Collembola	Onychiuridae	*Deuteraphorura kratochvili*	1451	
		Isotomidae	unidentif.	89	
			Folsomia candida	14	

	Neelidae	*Megalothorax*	11	
	Entomobryidae	*Heteromurus nitidus*	3	
	unidentif.		5	
Araneae	Linyphiidae	*Porrhomma profundum*	323	
Araneae	Linyphiidae	*Porrhomma profundum*	424	105877
Mesostigmata	Parasitidae	*Parasitus loricatus*	117	
Collembola	Isotomidae	*Folsomia candida*	84	
Araneae	Linyphiidae	*Porrhomma profundum*	62	14379
Insecta unidentif.			36	
Collembola	Isotomidae	*Folsomia candida*	34	
	Entomobryidae	*Heteromurus nitidus*	22	
Mesostigmata	Veigaiidae		29	
Araneae	Linyphiidae	*Porrhomma profundum*	62	96944
Insecta unidentif.			36	
Collembola	Isotomidae	*Folsomia candida*	34	
	Entomobryidae	*Heteromurus nitidus*	22	
Mesostigmata	Veigaiidae		29	
Collembola	Entomobryidae	*Heteromurus nitidus*	285	59704
Araneae	Linyphiidae	*Porrhomma profundum*	180	
Insecta unidentif.			110	
Diptera	Sciaridae	*Bradysia* (*forficulata*?)	64	
Coleoptera	Staphylinidae	*Quedius mesomelinus*	61	
Mesostigmata	Veigaiidae		56	
Trombidioformes	Eupodidae		5	
Hymenoptera	Braconidae		180	196075
Araneae	Linyphiidae	*Porrhomma profundum*	105	
Mesostigmata	Parasitidae	*Parasitus loricatus*	31	
Collembola	Isotomidae	*Parisotoma notabilis*	27	
Collembola	Entomobryidae	*Heteromurus nitidus*	584	119340
Insecta unidentif.			337	
Diptera	Sciaridae	*Bradysia* (*forficulata*?)	391	

	Araneae	Linyphiidae	*Porrhomma profundum*	36	
	Collembola	Neelidae	*Megalothorax*	211	191202
		Entomobryidae		67	
		Isotomidae		48	
		Neelidae	*Megalothorax*	13	
		unidentif.		8	
	Araneae	Linyphiidae	*Porrhomma profundum*	386	59410
	Mesostigmata	Laelapidae		313	
		Veigaiidae		195	
		Parasitidae	*Parasitus loricatus*	42	
	Collembola	Neelidae	*Megalothorax* sp. 1	36	
			Megalothorax sp. 2	3	
		unidentif.		22	
	Sarcoptiformes	Tectocepheidae?		3	
	Collembola	Entomobryidae	*Heteromurus nitidus*	169	74909
	Trombidioformes	Eupodidae		134	
	Araneae	Linyphiidae	*Porrhomma profundum*	49	
	Mesostigmata	Parasitidae	*Parasitus loricatus*	37	11397
	Collembola	Neelidae	*Megalothorax*	12	
		Mesogastruridae	*Mesogastrura*	16	
	Mesostigmata	Parasitidae		2	
	Palpigradi	Eukoeneniidae	*Eukoenenia spelaea*	0	0
	Collembola	Entomobryidae	*Heteromurus nitidus*	78	88426
	Araneae	Linyphiidae	*Porrhomma profundum*	11	
	Mesostigmata	Laelapidae		2	
	Diptera	Sciaridae	*Corynoptera*	3	23920
	Mesostigmata			2	
	Mesostigmata	Parasitidae	*Parasitus loricatus*	3	37739
		Veigaiidae		2	
Eukoenenia spelaea	Araneae	Linyphiidae	*Porrhomma profundum*	1302	0
	Trombidioformes	Eupodidae		454	

	Coleoptera	Staphylinidae	*Quedius mesomelinus*	210	
	Mesostigmata	Laelapidae		2	
				0	0
	Mesostigmata	Laelapidae		15856	0
	Collembola			10655	
	Araneae	Linyphiidae	*Centromerus cavernarum*	1066	
			Porrhomma profundum	3	
	Coleoptera	Staphylinidae	*Quedius mesomelinus*	19	
	Diptera	Cecidomyiidae		279	0
	Coleoptera	Staphylinidae	*Quedius mesomelinus*	15	
	Araneae	Linyphiidae	*Porrhomma profundum*	9	
	Collembola	Symphypleona		8	
	Araneae	Linyphiidae	*Centromerus cavernarum*	1030	0
			Porrhomma profundum	319	
			unidentif.	28	
	Mesostigmata	Parasitidae	*Parasitus loricatus*	406	
	Coleoptera	Staphylinidae	*Quedius mesomelinus*	25	
	Trombidioformes	Eupodidae		1055	0
				292	
	Mesostigmata	Parasitidae	*Parasitus loricatus*	464	
	Araneae	Linyphiidae	*Porrhomma profundum*	315	
			Centromerus cavernarum	3	
	Coleoptera	Staphylinidae	*Quedius mesomelinus*	222	
	Araneae	Linyphiidae	*Porrhomma profundum*	159	0
			Centromerus cavernarum	2	
	Coleoptera	Staphylinidae	*Quedius mesomelinus*	3	
	Collembola	Entomobryidae		35	0
	Sarcoptiformes	Nanorchestidae	*Speleorchestes*	6	
	Araneae	Linyphiidae	*Centromerus cavernarum*	3	
Parasitus loricatus	Diptera	Phoridae	*Triphleba?*	5	45967
	Palpigradi	Eukoeneniidae	*Eukoenenia spelaea*	-	

Palpigradi	Eukoeneniidae	*Eukoenenia spelaea*	-	
Sarcoptiformes	Damaeidae	*Kunstidamaeus lengersdorfi*	24	21425
Acari			3	
Trombidiformes			9	18766
Palpigradi	Eukoeneniidae	*Eukoenenia spelaea*	-	
Mesostigmata	Parasitidae	*Parasitus loricatus*	1074	39568
	Laelapidae		15	
Sarcoptiformes	Tectocepheidae?		2	
Palpigradi	Eukoeneniidae	*Eukoenenia spelaea*	-	
Trombidiformes			3	22754
			3	
Palpigradi	Eukoeneniidae	*Eukoenenia spelaea*	-	
			0	26929
Palpigradi	Eukoeneniidae	*Eukoenenia spelaea*	-	16020
			0	15627
Sarcoptiformes			4	22540
Trombidiformes			4	
			2	
Diptera	Phoridae	*Triphleba antricola*	35	24665
		Triphleba?	2	
Collembola	Isotomidae	*Parisotoma notabilis*	2	
Sarcoptiformes			9	23086
Coleoptera	Staphylinidae	*Quedius mesomelinus*	2	
Mesostigmata	Parasitidae	*Parasitus loricatus*	2977	48467
Arachnida unidentif.			15	
Trombidiformes			3	
Trombidiformes			68	75910
			25	
			17	
			13	
Sarcoptiformes	Tectocepheidae?		6	

Palpigradi	Eukoeneniidae	*Eukoenenia spelaea*	-	
Mesostigmata	Parasitidae	*Parasitus loricatus*	2816	56329
Palpigradi	Eukoeneniidae	*Eukoenenia spelaea*	-	
Palpigradi	Eukoeneniidae	*Eukoenenia spelaea*	-	20731
Palpigradi	Eukoeneniidae	*Eukoenenia spelaea*	-	18328
Mesostigmata	Parasitidae	*Parasitus loricatus*	1197	92080
	Veigaiidae		9	
Coleoptera	Staphylinidae	*Quedius mesomelinus*	169	
Araneae	Linyphiidae	*Centromerus cavernarum*	37	
		Porrhomma profundum	31	
Sarcoptiformes	Acaridae?		9	
Palpigradi	Eukoeneniidae	*Eukoenenia spelaea*	-	
Mesostigmata	Parasitidae	*Parasitus loricatus*	4584	85666
Mesostigmata	Parasitidae	*Parasitus loricatus*	1809	85803
Coleoptera	Staphylinidae	*Quedius mesomelinus*	6	
Mesostigmata	Parasitidae	*Parasitus loricatus*	1282	101645
Coleoptera	Staphylinidae	*Quedius mesomelinus*	4	
Mesostigmata	Parasitidae	*Parasitus loricatus*	1195	37512
Trombidiformes			20	
Sarcoptiformes	Tectocepheidae?		20	
Coleoptera	Staphylinidae	*Quedius mesomelinus*	2	
Palpigradi	Eukoeneniidae	*Eukoenenia spelaea*		
Mesostigmata	Parasitidae	*Parasitus loricatus*	3487	159361
Trombidiformes			23	
			12	
Coleoptera	Staphylinidae	*Quedius mesomelinus*	3	
Mesostigmata	Parasitidae	*Parasitus loricatus*	1540	73994
Coleoptera	Staphylinidae	*Quedius mesomelinus*	4	
Palpigradi	Eukoeneniidae	*Eukoenenia spelaea*	-	
Mesostigmata	Parasitidae	*Parasitus loricatus*	2500	88011
Diptera	Sciaridae	*Bradysia* (*forficulata*?)	569	

			Bradysia	2	
	Araneae	Linyphiidae	*Porrhomma profundum*	487	
	Coleoptera	Staphylinidae	*Quedius mesomelinus*	462	
	Trombidiformes			53	
				30	
				6	
	Mesostigmata	Veigaiidae		27	
Quedius mesomelinus	Coleoptera	Staphylinidae	*Quedius mesomelinus*	268	91226
				0	0
				0	92162
	Mesostigmata	Parasitidae	*Parasitus loricatus*	2	89495
	Coleoptera	Staphylinidae	*Quedius mesomelinus*	234	91421
	Glomerida			9	
	Diptera	Phoridae	*Triphleba antricola*	2	
	Collembola	Entomobryidae	*Heteromurus nitidus*	2	
	Diptera	Phoridae	*Triphleba antricola*	785	211405
	Coleoptera	Staphylinidae	*Quedius mesomelinus*	195	
	Diptera	Phoridae	*Triphleba antricola*	5	136443
	Glomerida			5	
				0	105047
	Coleoptera	Staphylinidae	*Quedius mesomelinus*	482	134042
	Diptera	Sciaridae	*Corynoptera spoeckeri*	13	
	Collembola			12	
				0	220329
				0	137009
				0	90436
	Glomerida			3	104375
	Mesostigmata	Parasitidae	*Parasitus loricatus*	5	
	Diptera	Sciaridae	*Camptochaeta scanica*	5	378946
				0	135596
				0	111280

Table S2. List of potential prey collected in Ardovská cave and their relative abundance.

Order	Species (Family)	Relative abundance
Palpigradi	*Eukoenenia spelaea*	0.005
Araneae	*Centromerus cavernarum*	0.001
	Porrhomma sp.	0.002
Oribatida	*Ceratoppia bipilis*	0.010
	Damaeus gracilipes	0.001
	Kustidamaeus lengersdorfi	0.011
	Multioppia cf. *glabra*	0.004
Gamasida	*Cyrtolaelaps chiropterae*	0.001
	Cyrtolaelaps mucronatus	0.001
	Parasitus loricatus	0.012
	Veigaia nemorensis	0.001
	Vulgarogamasus oudemansi	0.001
Actinedida	(Rhagidiidae)	0.001
Isopoda	*Mesoniscus graniger*	0.100
Diplopoda	*Trachysphaera costata*	0.002
	(Trichopolydesmidae)	0.012
Collembola	*Ceratophysella armata*	0.001
	Ceratophysella bengtssoni	0.002
	Deuteraphorura kratochvili	0.005
	Heteromurus nitidus	0.027
	Lepidocyrtus lignorum	0.025
	Megalothorax incertus	0.001
	Mesogastrura ojcoviensis	0.002
	Neelus koseli	0.001
	Parisotoma notabilis	0.067
	Protaphorura armata	0.004
	Pseudosinella aggtelekiensis	0.018
	Pygmarrhopalites aggtelekiensis	0.009
	Pygmarrhopalites pygmaeus	0.167
Diptera	*Bradysia forficulata*	0.063
	Camptochaeta ofenkaulis	0.008
	Culex pipiens	0.001
	Heteromyza atricornis	0.004
	Trichocera regelationis	0.004
	Trichocera sp.	0.009
	Triphleba antricola	0.142
	(Sphaeroceridae)	0.119
Coleoptera	*Aleochara funebris*	0.004
	Aleochara kamila	0.001
	Atheta spelaea	0.004
	Catops longulus	0.060

Catops f. *fuliginosus*	0.001
Catops picipes	0.001
Cryptophagus spp.	0.031
Ocypus biharicus	0.001
Pterostichus niger niger	0.001
Ptinus sp.	0.001
Quedius mesomelinus	0.034
Rhizophagus perforatus	0.001
Coleoptera larvae	0.017

Table S3: Predators (blue) and potential prey (black) collected in the Ardovská Cave and their reference COI sequences.

Order	Family	Species	Access. No.
Palpigradi	Eukoeneniidae	*Eukoenenia spelaea*	MN906450
Araneae	Linyphiidae	*Porrhomma profundum*	MN906451
		Centromerus cavernarum	MN906452
	Tetragnathidae	*Meta menardi*	MN906453
Mesostigmata	Parasitidae	*Parasitus loricatus*	MN906455
Oribatida	Damaeidae	*Kunstidamaeus lengersdorfi*	MN906454
Isopoda	Mesoniscidae	*Mesoniscus graniger*	-
Diplopoda	Trichoplydesmidae	sp. unident.	MN906456
	Trachysphaeridae	*Trachysphaera costata*	-
Collembola	Arrhopalitidae	*Pygmarrhopalites aggtelekiensis*	MN906457
	Entomobryidae	*Heteromurus nitidus*	MN906458
		Pseudosinella aggtelekiensis	MN906459
	Isotomidae	*Folsomia candida*	MN906460
		Parisotoma notabilis	MN906462
	Neelidae	*Megalothorax minimus*	-
	Onychiuridae	*Deuteraphorura kratochvili*	MN906461
	Tullbergiidae	*Mesaphorura jirii*	-
Coleoptera	Staphylinidae	*Quedius mesomelinus*	MN906463
Diptera	Sciaridae	*Bradysia forficulata*	MN906464
	Trichoceridae	*Trichocera regelationis*	MN906465
	Phoridae	*Triphleba antricola*	MN906466
Lepidoptera	Geometridae	*Triphosa dubitata*	MN906467
